# FIB-4 First in the Diagnostic Algorithm of Metabolic-Dysfunction-Associated Fatty Liver Disease in the Era of the Global Metabodemic

**DOI:** 10.3390/life11020143

**Published:** 2021-02-14

**Authors:** Yoshio Sumida, Masashi Yoneda, Katsutoshi Tokushige, Miwa Kawanaka, Hideki Fujii, Masato Yoneda, Kento Imajo, Hirokazu Takahashi, Yuichiro Eguchi, Masafumi Ono, Yuichi Nozaki, Hideyuki Hyogo, Masahiro Koseki, Yuichi Yoshida, Takumi Kawaguchi, Yoshihiro Kamada, Takeshi Okanoue, Atsushi Nakajima, Japan Study Group of NAFLD (JSG-NAFLD)

**Affiliations:** 1 Division of Hepatology and Pancreatology, Department of Internal Medicine, Aichi Medical University, Nagakute, Aichi 480-1195, Japan; yoneda@aichi-med-u.ac.jp; 2 Department of Internal Medicine, Institute of Gastroenterology, Tokyo Women’s Medical University, Tokyo 162-8666, Japan; tokushige.ige@twmu.ac.jp; 3 Department of General Internal Medicine2, Kawasaki Medical School, Okayama 700-8505, Japan; m.kawanaka@med.kawasaki-m.ac.jp; 4 Department of Hepatology, Graduate School of Medicine, Osaka City University, Osaka 558-8585, Japan; rolahideki@med.osaka-cu.ac.jp; 5 Department of Gastroenterology and Hepatology, Graduate School of Medicine, Yokohama City University, Yokohama 236-0004, Japan; yoneda-ycu@umin.ac.jp (M.Y.); kento318@yokohama-cu.ac.jp (K.I.); nakajima-tky@umin.ac.jp (A.N.); 6 Department of Metabolism and Endocrinology, Faculty of Medicine, Saga University, Saga 840-8502, Japan; takahas2@cc.saga-u.ac.jp; 7 Loco Medical General Institute, Saga 840-8502, Japan; eguchiyu@cc.saga-u.ac.jp; 8 Division of Gastroenterology and Hepatology, Department of Internal Medicine, Tokyo Women’s Medical University Medical Center East, Tokyo 116-8567, Japan; ono.masafumi@twmu.ac.jp; 9 Department of Gastroenterology, National Center for Global Health and Medicine, Tokyo 162-8655, Japan; ynozaki@hosp.ncgm.go.jp; 10 Department of Gastroenterology, JA Hiroshima General Hospital, Hiroshima 738-8503, Japan; hidehyogo@ae.auone-net.jp; 11 Division of Cardiovascular Medicine, Department of Medicine, Graduate School of Medicine, Osaka University, Suita, Osaka 565-0871, Japan; koseki@cardiology.med.osaka-u.ac.jp; 12 Department of Gastroenterology and Hepatology, Suita Municipal Hospital, Osaka 564-8567, Japan; yu1yoshida@gmail.com; 13 Division of Gastroenterology, Department of Medicine, Kurume University School of Medicine, Kurume 830-0011, Japan; takumi@med.kurume-u.ac.jp; 14 Department of Advanced Gastroenterology & Hepatology, Graduate School of Medicine, Osaka University, Suita, Osaka 565-0871, Japan; ykamada@gh.med.osaka-u.ac.jp; 15 Hepatology Center, Saiseikai Suita Hospital, Osaka 564-0013, Japan; okanoue@suita.saiseikai.or.jp; 16 Japan Strategic Medical Administration Research Center (J-SMARC), Nagoya, Aichi 460-0011, Japan

**Keywords:** hepatic fibrosis, hepatocellular carcinoma, vibration-controlled transient elastography, nonalcoholic fatty liver disease, type 2 diabetes, metabolic-dysfunction-associated fatty liver disease, cardiovascular disease

## Abstract

The prevalence of obesity or metabolic syndrome is increasing worldwide (globally metabodemic). Approximately 25% of the adult general population is suffering from nonalcoholic fatty liver disease (NAFLD), which has become a serious health problem. In 2020, global experts suggested that the nomenclature of NAFLD should be updated to metabolic-dysfunction-associated fatty liver disease (MAFLD). Hepatic fibrosis is the most significant determinant of all cause- and liver -related mortality in MAFLD. The non-invasive test (NIT) is urgently required to evaluate hepatic fibrosis in MAFLD. The fibrosis-4 (FIB-4) index is the first triaging tool for excluding advanced fibrosis because of its accuracy, simplicity, and cheapness, especially for general physicians or endocrinologists, although the FIB-4 index has several drawbacks. Accumulating evidence has suggested that vibration-controlled transient elastography (VCTE) and the enhanced liver fibrosis (ELF) test may become useful as the second step after triaging by the FIB-4 index. The leading cause of mortality in MAFLD is cardiovascular disease (CVD), extrahepatic malignancy, and liver-related diseases. MAFLD often complicates chronic kidney disease (CKD), resulting in increased simultaneous liver kidney transplantation. The FIB-4 index could be a predictor of not only liver-related mortality and incident hepatocellular carcinoma, but also prevalent and incident CKD, CVD, and extrahepatic malignancy. Although NITs as milestones for evaluating treatment efficacy have never been established, the FIB-4 index is expected to reflect histological hepatic fibrosis after treatment in several longitudinal studies. We here review the role of the FIB-4 index in the management of MAFLD.

## 1. Introduction

Obesity-associated disease is the most serious health problem worldwide (so-called metabodemic) [1]. In the adult population, 25% of the general population is estimated to be suffering from nonalcoholic fatty liver disease (NAFLD) [2]. Lifestyle-related diseases, such as obesity, type 2 diabetes (T2D), dyslipidemia, and hypertension, are closely associated with NAFLD, and patients who are not obese can also present with NAFLD; this is known as lean NAFLD. Lean NAFLD is defined as NAFLD that develops in patients with a body mass index (BMI) of <25 kg/m^2^ [3]. The prevalence of lean NAFLD varies from 7% in the US [4] to as high as 19% in Asia [5,6]. The pathogenesis of lean NAFLD is not well understood. Lean NAFLD patients demonstrate early alterations in the bile acid and gut microbiota profile [7]. The possession of the patatin-like phospholipase domain containing 3 (PNPLA3) polymorphism has been shown to be an independent factor associated with lean NAFLD patients [8,9].

The nomenclature of NAFLD should be updated to metabolic-dysfunction-associated fatty liver disease (MAFLD) [10]. Global experts suggest that the term MAFLD is more appropriate than NAFLD [11]. NAFLD has been diagnosed after exclusion of other liver diseases, while MAFLD can coexist with other liver diseases [10]. Therefore, MAFLD plus the hepatitis B virus (HBV) inactive carrier, MAFLD plus alcoholic liver disease (ALD), MAFLD plus autoimmune hepatitis (AIH), or MAFLD plus drug-induced liver injury (DILI) are plausible as a final diagnosis in clinical practice. Hepatic fibrosis is the most important risk factor for not only incident HCC, but also liver-related mortality in MAFLD [12]. Liver biopsy is now the gold standard for evaluating hepatic fibrosis, but it has several drawbacks such as hemorrhage risk, invasiveness, cost, observers’ variability, and patients’ unwillingness. Considering a large population of MAFLD patients, non-invasive tests (NITs) without performing liver biopsy are urgently required [13]. The American Association for the Study of Liver Disease (AASLD) practice guidance 2018 recommends the use of an NAFLD fibrosis score (NFS), the fibrosis-4 (FIB-4) index, vibration-controlled transient elastography (VCTE), and magnetic resonance elastography (MRE) [14]. However, all institutions do not have these innovative imaging modalities such as VCTE or MRE. The FIB-4 index, consisting of four parameters (age, aspartate aminotransferase (AST), alanine aminotransferase (ALT), and platelets), is a simple, cheap, and accurate tool [15,16]. We here review the role of the FIB-4 index for evaluation of hepatic fibrosis, incident comorbidities, carcinogenesis (hepatocellular carcinoma (HCC) and extrahepatic malignancy), overall/liver-related mortality or morbidity, and treatment efficacy in the management of NAFLD.

## 2. Which Fibrosis Stage Should We Pick up in MAFLD?

For a long time, a variety of NITs have been proposed to differentiate steatohepatitis from simple steatosis: cytokerarin-18, The hypertension, ALT levels, and insulin resistance (HAIR) score, and the NAFIC score (NASH, ferritin, insulin, and type IV collagen 7s). None of the NITs were globally accepted, because histological diagnosis of steatohepatitis has several limitations such as existence of borderline steatohepatitis, observers’ variability, and sampling error.

Fibrosis stages in MAFLD can be classified into F0, F1, F2, F3, and F4 [17,18]. F3 or F4 were defined as advanced fibrosis. Currently, MAFLD patients with advanced fibrosis should be examined for HCC surveillance considering cost-benefit balance [19,20]. Considering exponential increase in liver-related mortality in MAFLD patients with ≥ F2 compared with those with F0/1 (hazard ratio [HR] 9.57, 95% confidence interval [CI] 1.67–54.93) [12], we wonder which fibrosis stage (F2, F3, or F4) we should mine among a huge population of MAFLD. A variety of NITs for identifying advanced fibrosis in MAFLD have been established (Table 1). Vilar-Gomez et al. reported that NFS and the FIB-4 index are useful screening tools for determining the stage of liver fibrosis to be routinely applied in clinical practice [21]. Thus, the FIB-4 index and NFS are now recommended for excluding advanced fibrosis in the AASLD practice guidance 2018 [14].

The enhanced liver fibrosis (ELF) test is a non-invasive blood test that measures three direct markers of fibrosis: Hyaluronic acid (HA), procollagen III amino-terminal peptide (PIIINP), and tissue inhibitor of matrix metalloproteinase 1 (TIMP-1) [22]. According to a two-step algorithm from EU [23], ELF test can be applied to the intermediate group of FIB-4 index (1.3–3.25). If NAFLD patients have an ELF score of 10.35 or above, they are likely to have advanced fibrosis. ELF can reduce unnecessary liver biopsies. Recently, the usefulness of the ELF test was also validated in the Japanese NAFLD population [24]. Combinations or sequential procedures using VCTE complement the diagnostic performance of the ELF test for the identification of advanced fibrosis. From the view of economic cost, the combination of FIB-4 index plus ELF test is superior to the combination of FIB-4 index plus VCTE [25]. In the two-step algorithm for identifying severe fibrosis in MAFLD, FIB-4 index has been established as the 1st step, while ELF score, VCTE, or MRE may be diagnostic modalities as the 2nd step.

**Table 1:** A variety of non-invasive tests (NITs) for identifying severe fibrosis (F3/4) in MAFLD.

Index	Formula	Strengths	Weaknesses
FIB-4 index [15,16]	(age [years] × AST [U/L]/(platelet count [10^9^/L] × √ALT [U/L]) https://www.eapharma.co.jp/medicalexpert/product/livact/fib-4/calculator.html (accessed on 25 January 2021)	Simple (only four parameters) Accurate Validated globally	Requires an intermediate group Overpredict in old patients Inferior in patients with T2D?
NAFLD fibrosis score [26]	−1.675 + 0.037 × age (years) + 0.094 × BMI (kg/m^2^) + 1.13 × impaired fasting glucose/diabetes (yes = 1, no = 0) + 0.99 × AST/ALT ratio–0.013 × platelet count (×10^9^/L) − 0.66 × albumin (g/dL) http://nafldscore.com/ (accessed on 25 January 2021)	Validated globally Accurate	Complex (six parameters) Requires an intermediate group Overpredict in old patients
APRI [27]	AST to platelet ratio index	Simple (only two parameters)	Conflicting results
BARD [28]	BMI > 28 kg/m^2^ = 1 point AST/ALT ratio > 0.8 = 2 points Diabetes = 1 point	Very simple	Conflicting results
CA-fibrosis index [29]	1.5 × type IV collagen 7S (ng/mL) + 0.0264 × AST (IU/l)	Simple (only two parameters)	Only available in Japan No external validation studies
ELF test [22]	−7.412 + (In [HA] × 0.681) + (In [P3NP] × 0.775) + (In [TIMP1] × 0.494)	Accurate Validated globally	High cost? (three parameters)

MAFLD: Metabolic dysfunction-associated fatty liver disease, APRI: AST to platelet ratio index, BARD: BMI, AST/ALT ratio, and diabetes, ELF: Enhanced liver fibrosis, FIB-4: Fibrosis-4, AST: Aspartate aminotransferase, ALT: Alanine aminotransferase, BMI: Body mass index, HA: Hyaluronic acid, PIIINP: Aminoterminal propeptide of type III procollagen. TIMP-1: Tissue inhibitor of matrix metalloproteinase type 1, CA: Type IV collagen 7s and AST.

## 3. The Usefulness of FIB-4 Index to Evaluating Severe Fibrosis in MAFLD

The FIB-4 index is a score based on readily available blood tests that are routinely measured (age, AST, ALT, and platelet count). FIB-4 index is originally developed for evaluating hepatic fibrosis in patients with HIV/HCV co-infection [30]. At first, multiple regression analysis identified four variables as independent predictors of fibrosis: Age, AST, PT-INR, and platelet count in 505 patients with HIV/HCV co-infection. The second model that was investigated was applicable to 553 patients, and considered age, AST, platelet count, and ALT instead of PT-INR [30]. FIB-4 index enabled the correct identification of patients with severe fibrosis (F3/4) in HCV-monoinfected patients [31]. HCV eradicated patients without cirrhosis, but those with FIB-4 scores ≥ 3.25 have a high enough risk to merit HCC surveillance [32]. In noncirrhotic patients with chronic HBV infection, low FIB-4 index is useful for the prediction of the lowest risks of liver related events (carcinogenesis, cirrhosis progression, and mortality) [33,34,35]. Taken together, FIB-4 index has been established as NIT for identifying severe fibrosis or high risk of liver-related event in patients with chronic viral hepatitis.

In MAFLD, the first report by Shah and colleagues in a study of 541 MAFLD patients found that FIB-4 index had better diagnostic accuracy for estimation of liver fibrosis among various serum markers [15]. FIB-4 index has been suggested as a prescreening strategy to improve the efficiency of referral for specialized liver care, prioritizing patients who are at higher risk of significant liver disease. First of all, diagnostic accuracy is superior to other simple NITs such as NFS, AST to platelet ratio index (APRI), and BARD (BMI, AST/ALT ratio, diabetes) score [15,16,26,36,37,38,39,40] (Table 2). The NPV values of all methods (APRI, FIB-4 index, BARD score and NFS) were greater than 75% for the diagnosis of severe fibrosis. The summary specificities of the four models (APRI, FIB-4 index, and NFS) were greater than 85% for predicting severe fibrosis. The BARD score was inferior to other parameters. When APRI and FIB-4 index were used to detect severe fibrosis, their corresponding summary specificities were greater than 95%. The summary specificities of APRI (cutoff of 1.5), FIB-4 index (cutoff of 2.67), BARD score (cutoff of 2), and NFS (cutoff of 0.67–0.676) were 96.1%, 96.5%, 61.3%, and 94.6%, respectively. Only FIB-4 and NFS had a summary PPV greater than 70% [36].

FIB-4 index could differentiate between steatohepatitis and non-steatohepatitis, even with steatohepatitis patients with mild or no fibrosis [41]. FIB-4 index has several advantages. First, calculation of FIB-4 index requires only four parameters, age, AST, ALT, and platelet count, while calculating the formula of NFS is slightly more complex [26] (Table 1). Second, FIB-4 index is available even in MAFLD patients with normal ALT levels [42,43,44]. A meta-analysis proved that 25% MAFLD patients and 19% NASH patients possess the normal ALT value [43]. Another strength of FIB-4 index is the availability of free online calculators (https://www.eapharma.co.jp/medicalexpert/product/livact/fib-4/calculator.html) (accessed on 25 January 2021).

## 4. The Compassion between FIB-4 Index and VCTE

To assess liver fibrosis, several non-invasive US-based elastography techniques have been developed. These methods include VCTE (FibroScan; Echosens, Paris, France), acoustic radiation force impulse (ARFI) imaging, and shear wave elastography (SWE) [45]. US-based VCTE performed with the FibroScan (Echosens) is the most thoroughly validated and commonly used elastography method worldwide. A systematic review and meta-analysis of VCTE in patients with NAFLD by Kwok et al. indicated that VCTE is good for the diagnosis of F3 (85% sensitivity and 82% specificity) and excellent for F4 (92% sensitivity and 92% sensitivity). However, it has a slightly lower accuracy for diagnosing F2 (79% sensitivity and 75% specificity) [46]. VCTE has several limitations. VCTE is limited to referral centers due to high equipment cost and had substantial failure rate, especially in obese patients. VCTE has a better diagnostic accuracy for advanced fibrosis than both FIB-4 index and NFS only in nonobese and/or low ALT patients [47]. However, liver stiffness measurement (LSM) by VCTE is influenced by not only hepatic fibrosis, but also a various factors, including steatosis, inflammation, congestion, and cholestasis. LSM has also intra- or inter-observers’ variability. The two-step algorithm, using FIB-4 index as the first step followed by VCTE as the second step, has been proposed in the US, Canada, and Asia [48,49,50,51,52]. The optimal cutoff value of LSM for identifying advanced fibrosis should be discussed.

## 5. FIB-4 Index and Carcinogenesis

In HCV, increased risk for HCC persists up to 10 years after HCV eradication in patients with baseline cirrhosis or high FIB-4 index [53]. In hepatis virus infected patients, a meta-analysis confirmed prognostic values of the FIB-4 index for overall survival and recurrence-free survival in HCC [54]. In NAFLD, Kanwal et al. showed that an FIB-4 index > 2.67 is associated with an increased risk of HCC not only in those with known cirrhosis, but also in those without a prior diagnosis of cirrhosis [55]. It is noteworthy whether FIB-4 index can be a predictor of incident malignancy in NAFLD, including HCC. NAFLD patients had a higher risk of HCC, colon cancer, and breast cancer compared with the non-NAFLD population [56]. NAFLD patients with FIB-4 index > 1.45 had higher risk of all cancer incidence compared to those with FIB-4 index < 1.45 (HR: 13.99, 95% CI: 3.00–65.23) [56]. In another study, FIB-4 index and NFS can predict HCC development and extra-cancer incidence, although the number of NAFLD patients involved in this study is small (*n* = 123) [57]. In Japan, the FIB-4 index was useful for predicting liver-related diseases but had limitations in predicting extrahepatic malignancies [58]. The relationship between NITs and extrahepatic cancer should be explored further.

It remains to be solved whether hepatic fibrosis could accelerate carcinogenesis in extrahepatic organs.

## 6. FIB-4 Index and Mortality

NAFLD patients with higher FIB-4 index are associated with increased liver disease and overall mortality [59,60,61,62] (Table 3). When NITs are applied to the general population, NITs did not become better predictor of severe liver disease than expected [57]. In NAFLD with diabetes, FIB-4 index, NFS, and APRI cannot predict liver-related mortality and morbidity [63]. In Japan, liver related mortality is extremely low in US-diagnosed NAFLD patients (9/4073) [64]. The main cause of mortality in that study is cardiovascular events and extrahepatic malignancies. NFS can stratify risk of cardiovascular events and extrahepatic malignancies [64]. FIB-4 index is also associated with all-cause mortality of systemic chronic diseases such as rheumatoid arthritis [65], microscopic polyangiitis, granulomatosis with polyangiitis [66], and chronic obstructive pulmonary disease [67]. The underlying mechanisms of these relationships remain unknown.

## 7. FIB-4 Index and Risk of Cardiovascular Disease

The leading cause of mortality in MAFLD patients is cardiovascular disease (CVD), followed by extrahepatic cancer and liver related diseases [70]. MAFLD is an independent risk factor of coronary sclerosis [71], atrial fibrillation (AF) [72], coronary artery disease (CAD), and left ventricular dysfunction [73,74]. In daily clinical practice, we should pay attention to CVD event and control other risk factors, such as hypertension, dyslipidemia, and type 2 diabetes (T2D). FIB-4 index appears to be associated with high risk of CVD mortality [60]. Over a median follow-up time of 41.4 months (3044.4 patient-years) in 898 consecutive outpatients (mean age, 56.4 ± 12.7 years; 37.5% women), 58 cardiovascular events (1.9%/year) were registered. The rate of cardiovascular events was higher in patients with (*n* = 643, 2.1%/year) vs. without MAFLD (*n* = 255, 1.0%/year) (*p* = 0.066). In multivariable Cox proportional regression analysis, MAFLD increased risk for cardiovascular events (HR, 2.41; 95% CI, 1.06–5.47; *p* = 0.036) after adjustment for metabolic syndrome. Among patients with MAFLD, male sex, previous cardiovascular events, metabolic syndrome, and FIB-4 index ≥ 2.67 (HR, 4.02; 95% CI, 1.21–13.38; *p* = 0.023) were independently associated with risk of incident cardiovascular events [75]. A post hoc analysis of SAKURA AF Registry study showed that higher FIB-4 index ≥ 2.51 is independently associated with risks of CVD events and all-cause mortality in patients with AF [76]. The highest levels of NIT such as NFS, FIB-4 index, APRI, gamma-glutamyltransferase (GGT) to platelet ratio (GPR), and Forns score were associated with all-cause mortality and cardiovascular mortality [77]. In Japan, FIB-4 index is well correlated with coronary atherosclerosis (coronary artery calcium [CAC] score > 100), and subjects with higher FIB-4 index were prone to receive percutaneous coronary intervention [78]. In 665 Korean NAFLD subjects, the NFS and FIB-4 index were associated with coronary atherosclerosis (CAC score > 100) [79]. In patients with CAD, the highest NITs of hepatic fibrosis are associated with increased risks of all-cause and cardiovascular mortality [80]. FIB-4 index is also associated with all-cause mortality in patients with heart failure (HF) [81]. Among 96,373 participants over 6.9 years, 3844 incident congestive heart failure (CHF) events occurred. FIB-4 between 1.45 and 3.25 and FIB-4 > 3.25 were associated with incident CHF (HR [95% CI], 1.17 [1.07–1.27], and 1.65 [1.43–1.92], respectively) [82]. These results suggest that hepatic fibrosis (mild to severe) is associated with incident HF in the general population.

## 8. FIB-4 Index and Risk of Chronic Kidney Disease

MAFLD often complicates chronic kidney disease (CKD), resulting in growing indication for simultaneous liver kidney transplantation (SLKT) [83]. Risk of kidney graft loss was over 1.5-fold higher in recipients with MAFLD-cirrhosis than those with other etiologies [83]. A meta-analysis by Musso from Italy showed that MAFLD was associated with an increased risk of prevalent (OR 2.12, 95% CI 1.69–2.66) and incident (HR 1.79, 95% CI 1.65–1.95) CKD. Advanced fibrosis was associated with a higher prevalence (OR 5.20, 95% CI 3.14–8.61) and incidence (HR 3.29, 95% CI 2.30–4.71) of CKD than non-advanced fibrosis [84]. A variety of common drug pipelines exists for MAFLD and CKD [85,86]. In a cross sectional study based on 755 patients with USA-based diagnosed MAFLD, high FIB-4 index (≥1.10) is associated with an increased risk of prevalent CKD. The area under the receiver operating characteristic curve (AUROC) was the greatest for FIB-4 index (0.750), followed by NFS (0.710), AAR (0.594), APRI (0.587), and BARD score (0.561). In an analysis of the National Health and Nutrition Examination Survey (NHANES) conducted in the USA between 1988 and 1994, FIB-4 index is the better predictor of an increased risk of prevalent CKD compared with NFS, BARD, and APRI score [87].

The annual rate of incident CKD in MAFLD patients is estimated to be about 1.2% [88]. Five factors of baseline low eGFR level (60–75 mL/min), aging, T2D, hypertension, and elevated GGT, increase the risk of the development of CKD [88]. High FIB-4 index is a significant risk factor for incident CVD, and patients with increased FIB-4 index showed larger reduction in eGFR compared with those with decreased FIB-4 index [89]. The association of PNPLA3 genotype with incident CVD is conflicting [89,90,91].

## 9. Distribution of FIB-4 Index in MAFLD Population

The distribution of FIB-4 index in a healthy general population remains unknown, while some reports showed the distribution of FIB-4 index in MAFLD population. A total of 1370 MAFLD patients (78.5%) exhibited a low cut-off index (COI) (<1.30), 357 (20.5%), exhibited an indeterminate COI (1.30–2.67), and 18 (1.0%) exhibited a high COI (>2.67) [92]. Among 5410 Japanese MAFLD patients who were diagnosed by health checkups, 87.4% exhibited low COI (<1.45), 12.1% exhibited an indeterminate COI (1.45–3.26), and 0.5% exhibited a high COI (>3.26) [93]. On data of 576 MAFLD with biopsy proven MAFLD from JSG-NAFLD, 336 (58.3%) exhibited low COI (<1.45), 31.4% exhibited an indeterminate COI (1.45–3.26), and 59 (10.2%) exhibited a high COI (>3.26) [16]. Distribution of FIB-4 index in MAFLD depends on population age, ethnics, and selection bias (population-based, hospital-based, or biopsy proven). We are now planning to clarify the distribution of FIB-4 index in a healthy general population undergoing health checkups or non-biased MAFLD population.

## 10. Drawbacks of FIB-4 Index

FIB-4 index is a simple, reliable, and cheap parameter. Because FIB-4 index shows a high negative positive value (NPV) for detecting advanced fibrosis, FIB-4 index is useful to exclude advanced hepatic fibrosis. However, the FIB-4 index has also several drawbacks [94].

First, FIB-4 index requires an intermediate group. NAFLD patients classified into that group have to receive other NITs or liver biopsies. After exclusion of no or mild fibrosis, 2nd step diagnosis should be applied to the intermediate group. In Europe, the ELF test is usually applied to this intermediate group [23]. In the US or Asia, VCTE has been inducted as the second step.

Second, the positive predictive value (PPV) for identifying advanced fibrosis is not so high, so the FIB-4 index cannot help us to pick up advanced fibrosis.

**Figure 1:**
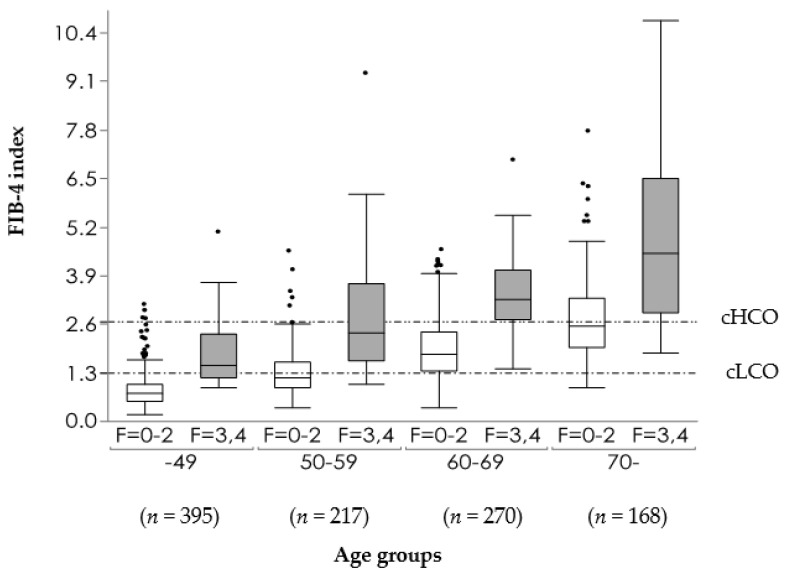
Box plot of the FIB-4 index in each age group according to the presence (F = 3, 4) or absence (F = 0–2) of advanced fibrosis [96]. cHCO: Conventional high cutoff, cLCO: Conventional low cutoff.

**Figure 2:**
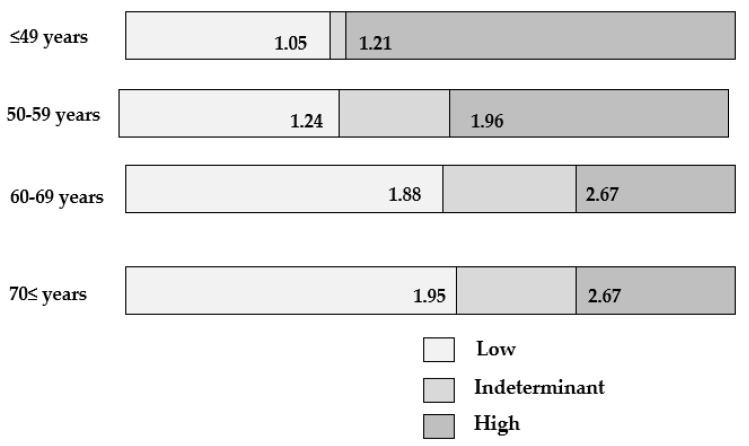
Modified cutoff values of FIB-4 index according to age group [96].

Third, there is a concern that FIB-4 index may overpredict fibrosis in older patients [95,96], because its formula includes age. On the basis of data in JSG-NAFLD including 1050 biopsy-proven MAFLD patients, the box plot of the FIB-4 index according to each age group was shown in Figure 1. The FIB-4 index increases with age. Using conventional COI, the exclusion of advanced fibrosis is decreasing as the age becoming higher, and the detection of advanced fibrosis is decreasing as the age become lower. The new proposed low COI are 1.88 in 60–69 years, and 1.95 in ≥70 years [96] (Figure 2). McPherson and colleagues also suggested 2.0 of low COI in 65 years or older [95]. On data of 1008 patients with MAFLD from nine centers across eight countries (The Gut and Obesity in Asia (GOASIA) Workgroup), NITs such as APRI, NFS, and FIB-4 index had a lower specificity in elderly (AUROC 0.62–0.65) [97]. Female (OR: 3.21; 95% CI 1.37–7.54] and hypertension (OR 3.68; 95%CI 1.11–12.23) were predicting factors for advanced fibrosis in the elderly [97].

Fourth, low COI of FIB-4 index are variable according to ethnics. Low COI of FIB-4 index was generally accepted as 1.3 in western countries [15,98], while 1.45 in Asia [16,50,52]. Over-referral and under-referral are tradeoff relationships (Table 4). The problem of over-referral includes increased unnecessary liver biopsies, overwork of hepatologists, and high healthcare costs [99]. Over-referral has merits, such as decrease in burden for general physician and early identification of HCC, resulting in improving overall survival. The selection of over-referral or under-referral depends on hospital human resources, and physicians‘ or hepatologists’ commitment for MAFLD.

Fifth, the FIB-4 index has limitations in a certain population of MAFLD patients. FIB-4 index showed significantly lower AUROCs for advanced fibrosis in obese MAFLD than in non-obese NAFLD [100]. Moreover, we found that FIB-4 index might be inferior in MAFLD patients with T2D compared to those without T2D [101]. In a study from Australia, NITs such as FIB-4 index, NFS, and APRI did not predict liver related events in 284 patients with MAFLD and diabetes [102]. Although its precise mechanism underlying inferiority of these NITs in T2D patients remains unknown, platelet count tends to be higher in MAFLD patients with T2D compared to those without T2D [101]. FIB-4 index in MAFLD patients with T2D is also lower than in those without T2D at the same fibrosis stages. FIB-4 index had reasonable specificity (69.9%), but poor sensitivity for detecting advanced fibrosis (72.6%) in T2D [103]. Type IV collagen 7S is the best predictor in Japanese MAFLD patients with T2D [101]. The combination of type IV collagen 7S and AST (CA index) may be more useful than type IV collagen 7S alone for detecting severe fibrosis [29].

Sixth, Shah S and colleagues feel that a low cut-off of 1.3 may be inappropriate, as it would include patients with F2 fibrosis [104]. They propose lowering COI of FIB-4 index to 1.0 in order to capture F2 patients. F2 fibrosis confers an increased mortality of liver-related diseases compared with no fibrosis (F0) (HR: 2.52) [12]. “Active fibrotic NASH” which requires intensive treatment is defined as NASH with NAFLD activity score (NAS) ≥ 4 and ≥ F2. Inclusion criteria in a variety of drug pipelines include NASH with NAS ≥ 4 and ≥ F2 [105,106]. FAST (FibroScan–AST) score, consisting of three parameters, including FibroScan-based controlled attenuation parameter (CAP), FibroScan-based LSM, and AST, can predict “active fibrotic NASH” [107,108,109]. “Active fibrotic NASH” patients had better receive intensive treatments for preventing progression to advanced stage. FAST score was designed to isolate “active fibrotic NASH” patients with elevated NAS ≥ 4 and significant fibrosis (≥F2) who could benefit from early interventions with anti-steatohepatitis and/or antifibrotic agents.

Although several problems of FIB-4 index remain to be solved, FIB-4 index is believed to be enough as the first triaging tool to exclude hepatic fibrosis, especially for general physicians or endocrinologists. However, limitations of FIB-4 index were kept in mind. As mentioned above, the MAFLD population with obesity or T2D might be inferior to that without obesity or T2D. It is plausible that heterogeneity of MAFLD has some impact on the performance of NIT.

$$\begin{array}{ll} \mathrm{FAST} & \mathrm{score}\;=\\ \; & \frac{e^{-1.65+1.07\times \mathrm{In}(\mathrm{LSM})+2.66\times 10^{-8}\times {\mathrm{CAP}}^{8}63.3\times \mathrm{AST}-1}}{1+e^{-1.65+1.07\times \mathrm{In}(\mathrm{LSM})+2.66\times 10^{-8}\times {\mathrm{CAP}}^{3}-63.3\times \mathrm{AST}-1}} \end{array}$$

## 11. Two-Step Diagnostic Algorithm Using FIB-4 Index as the First Step

**Figure 3:**
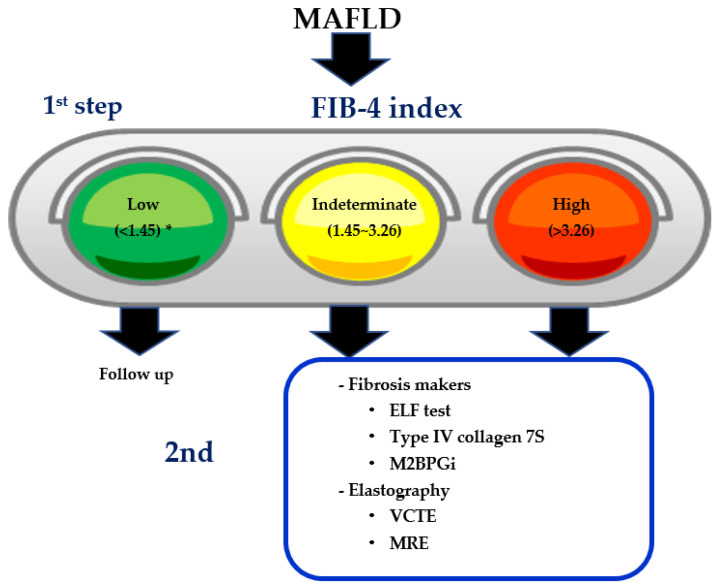
The two-step algorithm in MAFLD. * Higher low cutoff point (FIB-4 index < 2.0) can be applied to patients aged over 65 years. ELF: Enhanced liver fibrosis, M2BPGi: Mac-2 binding protein glycosylation isomer, VCTE: Vibration-controlled transient elastography, MRE: Magnetic resonance elastography.

Globally, two-step diagnostic algorithms using FIB-4 index as the first step are generally accepted. Assessment of the potential impact of implementing a FIB-4 first strategy to triage patients using a clinical referral pathway for suspected NAFLD was performed at a tertiary liver center in Canada [98]. FIB-4 first strategy would decrease costs and decrease unnecessary referrals, as well as increase access to screening in non-specialized facilities. It remains unknown which parameters are the most appropriate as the second step among a variety of NITs, including ELF test [22], Mac-2 binding protein glycated isomer (M2BPGi) [110,111,112], type IV collagen 7S [29,111], ProC3 [113], and autotaxin [114,115] (Figure 3).

## 12. FIB-4 Index as Milestones of Treatment in MAFLD

Hard endpoints of treatments such as overall or liver-related mortality are difficult to evaluate. The gold standard to evaluate steatohepatitis treatment efficacy is now histological finding by liver biopsy. The primary endpoints are (1) steatohepatitis resolution without worsening fibrosis, or (2) fibrosis improvement of more than 1 stage without worsening steatohepatitis. However, repeated biopsies are also difficult to perform, because of risk, patients’ unwillingness, cost, and diagnostic variability. NITs monitoring treatment efficacy are urgently needed to avoid repeated liver biopsies for evaluation of treatment efficacy. Hepatic steatosis has been evaluated by innovative imaging modalities such as VCTE-based CAP, magnetic resonance imaging-proton density fat fraction (MRI-PDFF), or ultrasound-guided attenuation parameter (UGAP) [116,117,118]. However, it remains unknown that reduction in hepatic fat content can really result in amelioration of hepatic fibrosis in MAFLD. It also remains unknown whether NITs evaluating hepatic fibrosis in cross-sectional studies can also reflect hepatic fibrosis in longitudinal studies. Accumulating evidence has suggested that improvement in ABC (ALT, body weight, and A1c) is related to ameliorating hepatic fibrosis [106]. It is expected that FIB-4 index can become alternative to liver biopsies for evaluating treatment efficacy [119,120]. Finally, reduction in ALT, body weight, HbA1c, APRI, and FIB-4 index may become milestones for ameliorate hepatic fibrosis in these longitudinal studies (Table 5).

## 13. Conclusions

In 2030, the number of Japanese MAFLD patients with advanced fibrosis are estimated to reach one million people. In China, about eight people will be suffering from advanced fibrosis. Early identification of advanced fibrosis can result in early detection of HCC or early intervention for MAFLD patients. The FIB-4 index is positioned as the first triaging tool for excluding advanced fibrosis due to its simplicity, low cost, and a predictor of liver-related or overall mortality. Type IV collagen 7S seems to be superior to the FIB-4 index in MAFLD patients with T2D. Type IV collagen 7S will become the first triaging tool for excluding advanced fibrosis in MAFLD patients with T2D. It remains unknown which NITs are the most appropriate as the second step in a two-step algorithm on the view of cost-benefit balance. The ELF test, VCTE, MRE, and other hepatic fibrosis markers are expected. 

We plan to examine two-step algorithm using FIB-4 first followed by type IV collagen 7S or M2BPGi. The FIB-4 index can predict incident CVD, CKD, and extrahepatic cancer. In clinical practice, repeated liver biopsies are difficult to perform in order to evaluate treatment efficacy. Accumulating evidence suggests that the FIB-4 index can also be used to evaluate treatment efficacy, although validation studies are required. It is concerned that primary care clinicians underestimate the prevalence of NAFLD and under-recognize the clinical spectrum of MAFLD. Interface between primary care and second care is essential for stratifying high risk of HCC/hepatic decompensation to improve survival in a large population of MAFLD. The FIB-4 index must help us to establish a referral pathway from primary care clinicians to hepatologists.

## Figures and Tables

**Table 2:** Summary sensitivities, specificities, PPV, and NPV of APRI, FIB-4, BARD score, and NAFLD Score, at various diagnostic thresholds for prediction of severe fibrosis [36].

Cutoff Values	No. of Studies (No. of Patients)	Summary Sensitivity, %, Mean (Range)	Summary Specificity, %, Mean (Range)	Summary PPV, %, Mean (Range)	Summary NPV, %, Mean (Range)
APRI					
0.452–0.50	5 (729)	72.9 (50.0–87.4)	67.7 (43.1–91.0)	44.8 (22.9–71.0)	89.4 (84.9–95.0)
0.54–0.98	7 (1,351)	68.6 (61.0–76.2)	72.7 (59.4–86.0)	61.4 (46.9–76.2)	77.6 (59.4–94.0)
1.00	4 (1101)	43.2 (27.0–67.0)	86.1 (81.0–89.0)	33.5 (26.0–40.0)	89.8 (84.0–95.0)
1.50	4 (682)	32.9 (6.3–70.0)	90.5 (74.5–97.0)	55.5 (40.0–72.1)	79.1 (73.2–87.2)
FIB-4 index					
1.24–1.45	10 (2759)	77.8 (63.0–90.0)	71.2 (55.5–88.0)	40.3 (24.0–50.6)	92.7 (88.0–98.0)
1.51–2.24	8 (1533)	77.0 (70.6–89.5)	79.2 (67.1–93.6)	66.4 (37.4–85.7)	83.9 (58.6–97.2)
2.67	6 (1910)	31.9 (12.0–63.2)	95.7 (88.3–98.7)	66.0 (51.1–80.0)	85.0 (79.4–92.6)
3.25	6 (1890)	37.3 (5.0–56.0)	95.8 (89.0–100)	72.5 (37.0–100)	87.3 (78.5–94.0)
5.31–10.62	4 (543)	67.5 (50.0–100)	80.8 (54.0–100)	90.0 (80.0–100)	85.1 (80.0–90.2)
BARD					
1.5	1 (242)	83.0	59.0	34.0	93.0
2	14 (3057)	75.2 (41.7–100)	61.6 (32.5–88.9)	38.3 (15.0–79.8)	88.7 (49.6–100)
3–4	5 (736)	59.4 (33.3–85.2)	75.1 (59.9–91.8)	55.2 (24.0–69.2)	81.0 (71.4–90.1)
NFS					
(−26.93)–(−2.16)	2 (106)	80.5 (78.0–83.0)	69.5 (69.0–70.0)	None	None
−1.455	10 (3057)	72.9 (22.7–96.0)	73.8 (42.9–100)	50.4 (24.0–100)	91.8 (81.3–98.1)
(−1.31)–(0.156)	5 (963)	78.2 (69.0–86.4)	71.7 (60.0–83.0)	58.4 (34.0–80.8)	82.1 (54.1–95.0)
0.67–0.676	14 (3896)	43.1 (8.3–100)	88.4 (25.0–100)	66.9 (26.0–100)	88.5 (78.6–100)
0.735	1 (235)	68.4	88.3	53.0	93.5

**Table 3:** NITs predicting for over-all mortality/morbidity, liver-related mortality/morbidity, liver related event, CVD, mortality, and extrahepatic cancer incidence in NAFLD.

Subjects	N	Nation	Dx	Observation Period	Over-all Mortality /Morbidity	Liver-Related Mortality/Morbidity	Liver Event	HCC	CVD Mortality	Extrahepatic Cancer
NAFLD [62]	646	Sweden	Biopsy	19.9 ±8.7 years	FIB-4 ○		FIB-4 ○			
					NFS ○		NFS ○			
Viral hepatitis-negative adults [61]	14,841	USA	General population	Median 19.3 years (IRQ, 17.5–21.1) years	APRI ○	APRI ○			FIB-4 ○	APRI ○
					FIB-4 ○	FIB-4 ○				
					NFS ○	NFS ○				
					Forns score ○	Forns score○				
NAFLD [57]	153	Israel	Biopsy	100 months (mean)	FIB-4 ○			FIB-4 ○		FIB-4 ○
					NFS ○			NFS ○		NFS ○
					APRI ×			APRI ○		APRI ○
NAFLD [68]	180	China	US	6.6 (range 0.5–14.8) years	NFS ◎					
					FIB-4 ○					
					APRI×					
					BARD×					
NAFLD [58]	646	Japan	Biopsy		FIB-4 ○			FIB-4 ○		FIB-4 ×
NAFLD [64]	4073	Japan	US						NFS ○	NFS ○
NAFLD with diabetes [63]	284	Australia	US	51.4 (range 6.1–146). months		NFS ×				
						FIB-4 ×				
						APRI ×				
NAFLD [60]	11,154	US	US	14.5 years	FIB-4 ○				FIB-4 ○	
					NFS ○				NFS ○	
					APRI ○				APRI ○	
NASH [69]	148	Canada	biopsy	Median: 5 years (IQR: 3–8)	FIB-4 ○					
					NFS ○					
					APRI ○					
NAFLD [59]	153	US	biopsy	Median 104.8 (range, 3–317) months		NFS ◎				
						FIB-4 ○				
						APRI ○				

HCC: Hepatocellular carcinoma, CVD: Cardiovascular disease, FIB-4: Fibrosis-4, NFS: NAFLD fibrosis score, APRI: AST to platelet ratio index, IQR: Interquartile range. Forns score = 7.811−3.131 log(platelet count [10^9^/L]) + 0.781 log(GGT [IU/L]) + 3.467 log(age [years]) − 0.014 total cholesterol (mg/dL) ◎ can predict very well, ○ can predict, × cannot predict.

**Table 4:** The tradeoff relationship between over-referral and under-referral for MAFLD.

	Over-Referral	Under-Referral
FIB-4 index low COI	1.3	1.45
GP	Work ↓	Work ↑
Hepatologists	Work ↑	Work ↓
Unnecessary liver biopsy	May increase	May reduce
HCC early detection	Possible?	May delay diagnosis?
Heath economic costs	High?	Low?

COI: Low cutoff index, GP: General physician, HCC: Hepatocellular carcinoma.

**Table 5:** NITs or parameters for monitoring treatment efficacy in MAFLD.

Author	Subjects	Outcomes	Parameter Correlated with Pathological Improvement
Hamaguchi [121]	MAFLD (*n* = 39)	Hepatic fibrosis	⊿HbA1c reduction
Seko [122]	Steatohepatitis (*n* = 52)	NAS Hepatic fibrosis	⊿ALT reduction ≥ 30% from baseline
Hoofnagle [123]	Steatohepatitis (*n* = 139) without DM PIVENS trial	NAS Hepatic fibrosis	⊿ALT reduction ≥ 30% from baseline or post-treatment ALT ≤ 40 IU/L
Vilar-Gomez [124]	Steatohepatitis (*n* = 261)	NASH resolution w/o worsening fibrosis	⊿BW reduction, absence of T2D ALT normalization, younger age, NAS < 5
Vuppalanchi	Adult steatohepatitis (*n* = 231) Pediatric MAFLD (*n* = 152)	Histological improvement	⊿CK18 reduction (inferior to ⊿ALT reduction)
Siddiqui [119]	MAFLD (*n* = 292)	Hepatic fibrosis	⊿FIB-4 index, ⊿NFS, ⊿APRI
Jayakumar [111]	Steatohepatitis, stage 2–3 (*n* = 54) Selonsertib (Phase 2)	Hepatic fibrosis	⊿MRE
		Hepatic steatosis	MRI-PDFF > 25% reduction
Chalasani [120]	Steatohepatitis (*n* = 200) FLINT trial (Phase 2) Placebo vs. OCA 72wk	Hepatic fibrosis	⊿FIB-4 index ⊿APRI (⊿NFS: no correlation)
Loomba		NAS ≥ 2 points reduction without worsening fibrosis	OCA(+), pretreatment NAS > 5, TG ≤ 154 mg/dL, INR < 1, AST < 49 IU/L, ⊿ALT at 24wk (>17 IU/L)

MAFLD: Metabolic dysfunction associated fatty liver disease, DM: Diabetes mellitus, HbA1c: Glycated hemoglobin, NAS: NAFLD activity score, ALT: Alanine aminotransferase, BW: Body weight, CK18: Cytokeratin 18, FIB-4: Fibrosis-4, NFS: NAFLD fibrosis score, APRI: AST to platelet ration, MRE: Magnetic resonance elastography, MRI-PDFF: Magnetic resonance imaging-proton density fat fraction, OCA: Obeticholic acid, NAS: NAFLD activity score, TG: Triglyceride, INR: International normalized ratio, AST: Aspartate aminotransferase, The ⊿ symbol represents the change in various laboratory values between the first and second liver biopsy.

## Data Availability

Permission for reuse of Figure 1 and Figure 2 was obtained from the licensed publisher (Springer Nature, License number:5006550258477) on 12 February 2021.

## Abbreviations

AASLD	American Association for the Study of Liver Diseases
AF	atrial fibrillation
AFP	α-Fetoprotein
AFP-L3	lens culinaris-agglutinin-reactive fraction of AFP
AGA	American Gastroenterology Association
AIM	apoptosis inhibitor of macrophage
AST	aspartate aminotransferase
ALD	alcoholic liver disease
ALT	alanine aminotransferase
APRI	AST to platelet ratio index
ARFI	acoustic radiation force impulse
AUROC	area under receiver operating characteristics curve
BMI	body mass index
HA	hyaluronic acid
PIIINP	aminoterminal propeptide of type III procollagen
TIMP-1	tissue inhibitor of matrix metalloproteinase type 1
CAC	coronary artery calcium
CAD	coronary artery disease
CAP	controlled attenuation parameter
CHF	congestive heart failure
CKD	chronic kidney disease
COI	cutoff index
CVD	cardiovascular disease
CI	confidence interval
CT	computed tomography
DILI	drug induced liver injury
eGFR	estimated glomerular filtration rate
ELF	enhanced liver fibrosis
ELISA	enzyme linked immunosolvent assay
FAST	FibroScan–AST
FIB-4	Fibrosis-4
GGT	gamma glutamyltransferas
HBV	hepatitis B virus
HCV	hepatitis C virus
HCC	hepatocellular carcinoma
HF	heart failure
HIV	human immunodeficiency virus
HR	hazard ratio
LSM	liver stiffness measurement
MAFLD	metabolism dysfunction associated fatty liver disease
M2BPGi	Mac-2 binding protein glycosylation isomer
MRE	magnetic resonance elastography
MRI	magnetic resonance imaging
NAFL	nonalcoholic fatty liver
NAFLD	nonalcoholic fatty liver disease
NASH	nonalcoholic steatohepatitis
NFS	NAFLD fibrosis score
NIT	non-invasive test
NPV	negative predictive value
OCA	obeticholic acid
OR	odds ratio
PDFF	proton density fat fraction
PIIINP	aminoterminal propeptide of type III procollagen
PNPLA3	patatin-like phospholipase domain-containing protein 3
PPV	positive predictive value
SLKT	simultaneous liver kidney transplantation
TIMP-1	tissue inhibitor of matrix metalloproteinase type 1,
T2D	type 2 diabetes
UCAP	ultrasound-guided attenuation parameter
US	ultrasonography
VCTE	vibration-controlled transient elastography
